# Modern Sociology

**Published:** 1899-12-23

**Authors:** 


### Modern Sociology.

### "PHOSSY JAW."

Dangerous trades cannot be entirely done away with, and therefore it is the more needful that they should be carried on under the most careful supervision. We cannot, unfortunately, rely on employers doing their best for the safety of their workers, and a comparatively recent prosecution has shown that some of them try to evade the rules laid down as to notifying cases of disease. The making of matches is a trade to which attention has frequently been drawn, but as yet it has not been found possible to wholly prevent the workers from suffering from that form of phosphorus poisoning which they themselves term "phossy jaw."

All workers in match factories do not suffer from this ailment. Those engaged in dipping the matches in the paste of which the heads are formed are the most liable to be affected, though those employed in other branches of the industry are by no means free from it. Moreover, some workers escape altogether, which shows that special conditions of health are necessary to induce infection. Now, this makes the evil difficult to deal with. There is hardly any industry which it would not be physically impossible for some persons to follow, and every year there must be many deaths due, directly or indirectly, to men and women taking up work for which they are unfit. But when a trade is admittedly dangerous it becomes the more incumbent on all connected with it to do their best to keep out workers whose health makes it unlikely they can stand the conditions of labour, and also to improve these conditions as far as possible. The Women's Trades Union League has issued a memorandum on the use of yellow phosphorus in the manufacture of matches which contains some useful suggestion on both points.

"Phossy jaw" is, in more scientific language, necrosis of the jaw. The poison attacks most readily persons whose teeth are decayed, and, therefore, it is desirable that anyone seeking employment in a match factory should have their teeth examined by a competent dentist before being accepted. This would at once eliminate a number of workers, and though these would grumble—for one of the chief difficulties experienced in protecting workers is their own indifference—it is probable that there would be an immediate decrease in the number whose jaws were affected. If it were possible to drive into the minds of those who still obtained employment the importance of keeping their teeth clean, even more would escape; but this is a counsel of perfection, and we do not expect it to be followed. It would be well, too, to keep out of the match factories all young persons under the age of eighteen. It is likely enough that their teeth might be in a better condition than those of their seniors, but it would be even more difficult to induce them to take care of them; and, moreover, growing young people are often more likely to take infection than those whose strength is not partly used up in the process of growth. There should, at least, be periodical medical inspection, so that the first indications of the disease might be observed and treated. Special tendencies, such as phthisis, syphilis, alcoholism, and anæmia, make those who suffer from them more liable to phosphorus poisoning; but, indeed, any debilitating conditions render infection easy. Among these a lack of sufficient nourishing food must be reckoned, and this makes any remedy difficult; for the work in match factories is not well paid, and a woman who is dependent on her own earnings, as we must assume the majority to be, is very unlikely to be able to keep herself in such a state of health as will enable her to resist the poison.

Something might be done by limiting the amount of yellow phosphorus to be used in the paste for the match-heads. In Belgium only 8 per cent. is allowed; and in Holland—at least in factories where women and young persons under 16 years of age are employed—only 5 per cent. of phosphorus is permitted. Some good might also come from not letting any person work at the most dangerous parts of the work for more than a limited time. In one place, workers are employed at dipping matches only for a fortnight at a time. The alternation of this work with other of a less dangerous kind would tend to maintain the general health of the workers, and enable them to resist the effects of the poison. Perhaps the appointment of a woman factory inspector would help the workers, so many of whom are women. Men inspectors look more to the general condition of things, where women are quick to note details, especially in the appearance of the workers. A bandaged face would attract a woman's attention more readily than it would a man's; and, also, an inquiry on the subject from a woman would not cause so much irritation as it would if it came from the other sex. In this, as in other trades, the rules as to providing conveniences for washing are not definite. There is no doubt that workers infect themselves by their neglect of cleanliness, but often the insufficiency of basins and towels provided for them is a comprehensible excuse. The Women's Trades Union League recommend that a basin and tap should be provided for every five workers, and, of course, that there should be a sufficient supply of water, and conveniences for emptying waste water on the spot. They also recommend the supplying of antiseptic tooth-powder or tooth-wash; but again we fear that the workers themselves would be slow in profiting by these conveniences. Special ventilation which should draw the fumes of the phosphorus away from the faces of the workers would also tend to diminish the risk of infection. Mr. Newlands, one of H.M.'s inspectors of factories, has invented a process which ensures this, but until the adoption of some such plan is made compulsory it will not be generally adopted.

And, after all, the only way to control a dangerous trade is to take the danger out of it. Can we not make matches without phosphorus? Are there no other substances to be obtained at a moderate cost which will give us all the results to be obtained from the use of phosphorus without doing any mischief to the workers? From many other trades which were once as full of danger to the workers as this better processes and the employment of new substances have taken away all risk. Let our chemists set themselves seriously to work to discover a substitute for yellow phosphorus, and we shall hear no more about "phossy jaw."

